# Gestational Weight Gain, Pregnancy Related Complications and the Short-Term Risks for the Offspring

**DOI:** 10.3390/jcm13020445

**Published:** 2024-01-13

**Authors:** Milan Lackovic, Milena Jankovic, Sladjana Mihajlovic, Zagorka Milovanovic, Marija Rovcanin, Nikola Mitic, Dejan Nikolic

**Affiliations:** 1 Department of Obstetrics and Gynecology, University Hospital “Dragisa Misovic”, Heroja Milana Tepica 1, 11000 Belgrade, Serbia; lackovic011@gmail.com (M.L.);; 2 Faculty of Medicine, University of Belgrade, 11000 Belgrade, Serbiadejan.nikolic@udk.bg.ac.rs (D.N.); 3 Neurology Clinic, University Clinical Center of Serbia, 11000 Belgrade, Serbia; 4 Clinic for Gynecology and Obstetrics “Narodni Front”, 11000 Belgrade, Serbia; 5 Department of Physical Medicine and Rehabilitation, University Children’s Hospital, 11000 Belgrade, Serbia

**Keywords:** gestational weight gain, excessive, pregnancy complication, offspring, early motor development

## Abstract

Background and objectives: Maternal obesity influences pregnancy course in several different manners, and imbalanced nutrition during pregnancy may lead to various adverse pregnancy outcomes. Additionally, nutritional status during pregnancy may have implications for the health of the offspring and may possibly influence early motor development in children. The aim of this study was to assess the impact of excessive gestational weight gain (EGWG) on pregnancy outcomes and infant’s motor development within the first twelve months of life. Materials and methods: The study included 200 participants divided in two groups based on their gestational weight gain. Maternal, perinatal, and neonatal factors were analyzed, and early motor development was assessed using the Alberta infant motor scale (AIMS). Results: EGWG was significantly associated with: pre-pregnancy BMI (*p* < 0.001), family history for cardiovascular diseases (*p* = 0.013) and diabetes mellitus (*p* = 0.045), hypertensive disorder of pregnancy (*p* = 0.003), gestational diabetes mellitus (*p* < 0.001), gestational anemia (*p* = 0.001), vitamin D deficiency (*p* = 0.001), metformin use (*p* = 0.045), pre-labor premature rupture of membranes (*p* = 0.031), amniotic fluid index (*p* = 0.047), and APGAR score in the first five min of life (*p* = 0.007). Scored by AIMS, EGWG was significantly associated with parameters of early motor development at the age of three AIMS total (*p* < 0.001), six AIMS total (*p* < 0.001), nine AIMS total (*p* < 0.001), and twelve AIMS total (*p* < 0.001) months of infant’s life. Conclusions: The link between EGWG and adverse neurodevelopmental outcomes in offspring is a complex and multifaceted issue. Our results imply significant alterations in early motor development in the group of infants born from mothers who gained weight excessively during pregnancy. Further studies are needed to unravel the intricacies of this relationship and inform strategies for preventive interventions and supportive care during pregnancy and infancy.

## 1. Introduction

Maternal obesity and excessive weight gain during pregnancy play a crucial role in pregnancy outcomes and affect maternal, fetal, and neonatal wellbeing in several ways [1,2]. An unbalanced diet during pregnancy, leading to inadequate or excessive weight gain, is associated with various adverse pregnancy outcomes. Gestational diabetes, high blood pressure during pregnancy, increased cesarean section rates, and altered fetal growth dynamics are among the most common consequences [3,4]. In addition, excessive weight gain during pregnancy can lead to long-term health problems in the mother, such as postpartum weight retention, obesity, postpartum depression, and metabolic syndrome [5]. Recent studies have indicated an increase in the prevalence of excessive gestational weight gain in the United States and Europe, and factors such as different ethnicities, socioeconomic status, and lifestyle have been identified as significant contributors [6,7]. 

For the developing fetus, excessive weight gain during pregnancy is associated with accelerated fetal growth dynamics, fetal macrosomia, which can eventually lead to birth injuries, complications during delivery, and an increased cesarean section rate [8]. Of even greater concern is the increased risk of the child developing obesity and other health problems later in life. Thus, lifestyle choices and actions taken during pregnancy appear to determine not only phenotypic and anthropometric characteristics, but also lifelong metabolic health outcomes and prospects for subsequent generations [9].

While weight loss during pregnancy is associated with a reduced risk of fetal macrosomia and cesarean section, weight loss during pregnancy is associated with low birth weight and has not been associated with significant improvements in perinatal outcomes [4]. Therefore, optimal nutritional requirements in pregnancy should be carefully considered and gestational weight goals should be set at the beginning of pregnancy according to pre-pregnancy body mass index categories. Maintaining a healthy weight during pregnancy is crucial for the wellbeing of both the mother and the developing fetus [10].

Research suggests that the nutritional status of the mother during pregnancy may also have an impact on the child’s early motor development. Adequate weight gain during pregnancy is associated with better neurodevelopmental outcomes, while inadequate or excessive weight gain may contribute to delays in the acquisition of motor skills [11]. Fetal macrosomia is often associated with excessive weight gain during pregnancy, and macrosomic infants may present with motor problems, such as delayed milestones and difficulty performing certain movements [12]. Conversely, inadequate weight gain during pregnancy can also have consequences, potentially leading to low birth weight and associated developmental problems [13]. Balancing and maintaining a healthy weight during pregnancy is therefore crucial for optimizing the motor development and general wellbeing of the newborn. Regular prenatal check-ups and advice from healthcare professionals play a crucial role in monitoring and managing weight gain during pregnancy [14].

Understanding the complex relationship between gestational weight gain, pregnancy complications, and early motor development is critical for healthcare providers to develop effective interventions that achieve optimal outcomes for both mothers and infants. Therefore, the aim of this study was to investigate the impact of excessive weight gain during pregnancy on pregnancy outcomes and infant motor development in the first twelve months of life.

## 2. Methods

### 2.1. Study Design

The clinical observational study lasted from August 2019 until January 2021, and it included 200 eligible “mother-infant” pairs randomly selected from the hospital computer database according to the maternal gestational weight gain values. All subjects in this study had regular pregnancy follow ups, and gave birth and had postnatal check-ups in the University hospital “Dr. Dragisa Misovic” in Belgrade, Serbia. Principles of good clinical practice and the Declaration of Helsinki were applied, and the study obtained the Institutional Review Board (IRB) (No. 01-14706/19, Date: 22 November 2019) approval. 

### 2.2. Study Participants 

The selection of study participants was based on gestational weight gain (GWG) by order, so that every fifth pregnant woman admitted to the obstetric ward was included up to a total of 200. The study group included 87 subjects who had gained excess weight, and the control group consisted of 113 subjects whose gestational weight gain met Institute of Medicine (IOM) recommendations [15].

Each participant’s weight before conception and at delivery was determined and the GWG was calculated as the mathematical difference between the two weights. The IOM recommendations for weight gain during pregnancy are dependent on pre-pregnancy body mass index (BMI) values. BMI values are calculated as weight in kilograms divided by height in meters squared. Based on the BMI values and the recommendation of the World Health Organization (WHO), four BMI categories are defined: Underweight (BMI < 18.5 kg/m^2^), normal weight (BMI between 18.5–24.9 kg/m^2^), overweight (BMI between 25.0–29.9 kg/m^2^), and obese (BMI > 30.0 kg/m^2^) [16]. Approximately 3.5 kg (kg) of total weight gain during pregnancy counts as fetal weight, while amniotic fluid is up to 1 kg and the weight of placenta is approximately 0.5 kg. Remaining weight gain counts as adipose tissue and extravascular fluid accumulation [17]. The IOM guidelines recommend a weight gain of 12.5–18.0 kg for underweight, 11.5–16.0 kg for normal weight, 7.0–11.5 kg for overweight, and 5.0–9.0 kg for obese pregnant women [15]. An increase in gestational weight beyond these ranges is considered excessive and was used as the cut-off point in this study.

### 2.3. Exclusion Criteria

Exclusion criteria were defined based on maternal age at the time of conception. All women aged <18 and >45 were excluded from the study, as were women with multiple pregnancies and chronic health issues. The presence of fetal or newborn defects or malformations were the exclusion criteria for the follow-up of the newborns from the study.

### 2.4. Study Variables 

All variables were divided into three study sets: maternal, perinatal, and neonatal and infant.

#### 2.4.1. Maternal Variables Included

Maternal age, pre-pregnancy and at delivery BMI, BMI category, gestational age, family history for cardiovascular disease (CVD) and diabetes mellitus (DM), hypertensive disorder of pregnancy (HDP), gestational diabetes mellitus (GDM), gestational anemia (GA), vitamin D deficiency, metformin use during pregnancy, genitourinary tract infection. 

#### 2.4.2. Perinatal Variables Included

Prelabor premature membrane rupture (PROM), fetal macrosomia, delivery mode, and delivery complications; obstetric ultrasound measurements included estimated fetal weight (EFW), amniotic fluid index (AFI), and fetal growth restriction.

#### 2.4.3. Neonatal and Infant Variables Included

APGAR score in the first and the fifth minute of life and infants motor development at the age of three, six, nine, and twelve months.

Reports from primary health center databases were used to determine pre-pregnancy BMI (based on pre-pregnancy weight and height), family history of cardiovascular disease, and diabetes mellitus. The American College of Obstetrics and Gynecology (ACOG) recommendations were used to define HDP [18], and 25-hydroxyvitamin D levels were assessed early in pregnancy to diagnose vitamin D deficiency [19]. The American Diabetes Association (ADA) recommendations were used to define GDM [20]. A hemoglobin concentration below 110 g/L was a diagnostic criterion for anemia in pregnancy [21]. Data on weight and BMI at delivery, genitourinary tract infections, and metformin use were obtained from the patients’ medical records. The first pregnancy ultrasound was performed between 6–8 weeks of pregnancy, the second between 12–14 gestational weeks, and, starting from the 24th gestational week, ultrasound was performed every two to four weeks. All ultrasound measurements were performed on the same model of ultrasound by the same sonographer. EFW was ascertained using biparietal diameter (BPD), head circumference (HC), abdominal circumference (AC), and femur length (FL). The fluid in the four quadrants of the uterus was measured to calculate AFI. EFW and AFI measured on the last perinatal ultrasound, no more than three days before the delivery, are presented in the tables below. Fetal macrosomia was defined as birth weight over 4000 g [22] and fetal growth restriction according to the Delphi consensus [23]. We studied four types of delivery: spontaneous vaginal delivery, cesarean section, assisted prostaglandin-induced labor, and cesarean section after failed induction of labor. Postpartum hemorrhage [24], retained placenta, uterine atony, and blood transfusion were considered as delivery complications. The motor development of the infants was assessed using the Alberta infant motor scale (AIMS). The AIMS consists of 58 items, including pronation (21), supination (9), sitting (12), and standing (16). It is a non-referenced measure with high specificity and sensitivity [25]. The AIMS test was performed by a trained assistant physician under the supervision of a specialist in physical medicine and rehabilitation.

### 2.5. Statistical Analysis

All statistical tests were performed with SPSS Statistics V.22.0. The results are presented as absolute (n) and relative (%) numbers, mean values (MVs), and standard deviation (SD). In addition, 95% confidence intervals (CIs) were introduced for continuous variables. Statistical significance was set at *p* < 0.05. Comparisons between the examined patient groups were performed using the Mann–Whitney U test for continuous variables and the chi-square test for categorical variables. A univariate regression model was used to determine potential risk factors from a set of maternal, perinatal, neonatal, and infant parameters in mothers with excessive weight gain during pregnancy.

## 3. Results

Our study included 200 subjects: 113 with normal range gestational weight gain (GWG) and 87 with excessive gestational weight gain (EGWG). EGWG patients had higher pre-pregnancy BMI (*p* < 0.001), pre-pregnancy BMI category (*p* < 0.001), and BMI at delivery (*p* < 0.001). Positive family history for CVD (*p* = 0.011) and DM (*p* = 0.038) was more common in the EGWG group of patients. Furthermore, more so than others, EGWG patients used metformin (*p* = 0.038) and had HDP (*p* = 0.001), GM (*p* < 0.001), GA (*p* < 0.001), VitD deficiency (*p* = 0.001), and PROM (*p* = 0.027). APGAR scores in the 1 min (*p* = 0.011) and the 5 min (*p* = 0.002) were lower in this group as well. Even though there were no statistically significant differences, some differences were observed in the delivery modes between the groups: cesarean section rates (16.81% vs. 22.99%, respectively), induction of labor (2.65% vs. 4.59%, respectively), and cesarean section after failed labor induction (4.42% vs. 8.04%, respectively) were higher, but not significantly, in the group of patients who gained excessively during pregnancy (Table 1).

The association of maternal, perinatal, and neonatal characteristics with EGWG is presented, in Table 2, by univariate logistic regression analysis. The EGWG was significantly associated with: pre-pregnancy BMI (*p* < 0.001), BMI category (*p* < 0.001), family history for CVD (*p* = 0.013) and DM (*p* = 0.045), HDP (*p* = 0.003), GDM (*p* < 0.001), GA (*p* = 0.001), VitD deficiency (*p* = 0.001), metformin use (*p* = 0.045), PROM (*p* = 0.031), AFI (*p* = 0.047), and APGAR score in the first 5 min of life (*p* = 0.007) (Table 2).

At the age of 3 months, infants born from EGWG mothers had lower AIMS pronation (*p* < 0.001), supination (*p* < 0.001), and total (*p* < 0.001) scores. At the age of 6 months, they had lower AIMS pronation (*p* < 0.001), supination (*p* < 0.001), sitting (*p* = 0.019), and total (*p* < 0.001) scores. Infants at the age of 9 months had lower AIMS pronation (*p* = 0.002), supination (*p* = 0.034), sitting (*p* < 0.001), standing (*p* = 0.001), and total (*p* < 0.001) scores. At the age of 12 months, there were no more differences in AIMS pronation and supination, but AIMS scores for sitting (*p* = 0.019), standing (*p* < 0.001), and total (*p* < 0.001) were lower (Table 3). 

The association between infants’ early motor development scored by AIMS and EGWG is presented, in Table 4, by univariate logistic regression analysis. The EGWG was significantly associated with: AIMS promotion (*p* < 0.001), supination (*p* < 0.001) and total (*p* < 0.001) score at the age of 3 months, AIMS promotion (*p* < 0.001), supination (*p* < 0.001) and total (*p* < 0.001) score at the age of 6 months, AIMS promotion (*p* = 0.001), supination (*p* = 0.042), siting (*p* < 0.001), standing (*p* = 0.001) and total (*p* < 0.001) score at the age of 9 months and AIMS siting (*p* = 0.041), standing (*p* < 0.001), and total (*p* < 0.001) score at the age of 12 months (Table 4).

In the Supplementary Material, we included multivariate logistic regression of tested parameters.

## 4. Discussion

In our study, 43.5% of the subjects had excessive GWG (EGWG). Patients who had excessive weight gain during pregnancy had higher pre-pregnancy and at delivery BMI, they were more likely to a have positive family history for CVD and DM, and developed various complications during pregnancy, including HDP, GDM, GA, and VitD deficiency. They were more likely to require metformin treatment more frequently and had higher AFI levels and PROM. Newborns born to mothers with EGWG had lower APGAR scores at the first five minutes of life, and we have observed significant differences in AIMS scores at the age of three, six, nine, and twelve months of life. 

It appears that pre-pregnancy weight and BMI category had a seminal impact on our patients’ commitment to a healthy lifestyle during pregnancy, because of the 87 subjects with EGWG, 14 were obese and 61 were overweight. Only 12 out of 87 patients with EGWG were normal weight, compared to 100 out of 113 subjects in the control group. Other authors also emphasize the role and importance of the interplay between pre-pregnancy overweight and obesity with unsatisfactory GWG goals [26], and underline the importance of weight reduction before becoming pregnant [27]. Weight is a modifiable risk factor associated with poor pregnancy outcomes [28], and preconception counselling, as the most effective primary prevention intervention, should provide nutritional guidance and support. Preconception counselling may minimize the overall risk for the poor pregnancy outcome and ensure a healthy environment for the fetal development by informing patients about the benefits of early prenatal care and optimal maternal health. Even though lifestyle interventions have the primary role, recent data suggest that approximately one-third of women may also be affected by behavioral disorders as well. Levine et al. discussed the importance of behavioral mechanisms and loss of eating control (LOC) in women who were overweight and obese before conception and found that prenatal LOC predicted EGWG [29]. LOC is associated with increased daily caloric intake and overeating episodes. It affects up to 30% of women of reproductive age, and up to 36% of women during pregnancy [30]. 

It is well known that pre-pregnancy overweight and obesity are risk factors associated with adverse pregnancy outcomes, but, not a long time ago, it was considered that EGWG has only limited influence on pregnancy outcomes [31]. The EGWG prevalence of 43.5% in our study is still not as high as the estimated EGWG prevalence in the United States [32], but it appears that the Serbian population, like the rest of the Europe [33], is slipping dangerously close to a pre-pregnancy obesity prevalence of 50%. The modern and stressful lifestyle, western diet, and lack of exercise seem to have led to an obesity pandemic in high-income countries around the world [34], from the United States to Australia, where the percentage share of pre-pregnancy overweight and obesity has reached endemic proportions and is now over 33% [35]. Therefore, more resources, funding, and public exposure should be directed to educational purposes in order to moderate the exponential obesity growth trend and mitigate the consequences of obesity among women of reproductive age. 

EGWG was a risk factor for some of the most serious pregnancy-related complications in our study, such as HDP and GDM. It is a well-known fact that the most common complications associated with maternal obesity are HDP and GDM, two major factors contributing to maternal morbidity and mortality [2,36]. Along with postpartum hemorrhage, HDP is the leading cause of maternal mortality in developed countries and the most devastating outcome in obstetrics [37]. Despite all diagnostic and therapeutic improvements, the management of HDP can be very challenging and has certain limitations [38]. Interrelationship between pre-pregnancy BMI, IOM recommended weight, and the HDP risk should be carefully analyzed by an obstetrician at each perinatal visit, and patients should be clearly informed that EGWG is undoubtedly associated with an increased risk of HDP [39]. According to the findings of Macdonald-Wallis et al. [40] measures implemented early in pregnancy to prevent EGWG are effective in reducing the risk of HDP. Although quite common, GA is another condition requiring early intervention and supplementation during pregnancy, especially among pregnant women dealing with EGWG. Untreated GA may lead to significant perinatal morbidity, and the etiology of GA must be clarified early in pregnancy [41]. The likelihood of GA in our study was 3.5 times higher in the EGWG group of patients, which indicates the extent of this condition and necessity for prompt treatment.

The diagnosis of GDM was made in 51 cases, and the prevalence of GDM in the EGWG group was more than 70%. The high prevalence of GDM associated with EGWG is also consistent with the findings of other authors [42], and to optimize pregnancy outcome in patients with GDM, control of GWG must be a priority [43]. In cases where diet failed to improve maternal glycemic control, we introduced metformin therapy. Eighteen subjects were prescribed metformin during pregnancy, twelve in the EGWG group and six in the control group. Metformin use during pregnancy is increasing due to high rates of maternal obesity, GDM, and type 2 diabetes, and even though metformin use appears to be safe and effective, its long-term effects on the offspring are still debatable and further research is needed [44]. It has been reported that metformin use during pregnancy may lead to lower birth weight [45], and our data may indeed confirm such assumptions. GDM occurred significantly more frequently in the EGWG group, but surprisingly there were no differences in macrosomia prevalence between the groups, even though neonates in the EGWG gained on average 100 g more. Vitamin D (VitD) deficiency may be another factor associated with higher GDM prevalence in the EGWG group, and we did find significant differences in VitD deficiency prevalence between the groups. Shao et al. analyzed correlation between maternal obesity, VitD deficiency, and GDM, and found a much greater GDM risk among obese pregnant women deficient in VitD [46]. Pantovic et al. reported that more than 60% of apparently healthy individuals in Serbia were deficient in vitD, with intake that was far below recommended values; they also found an inverse association between vitD status and obesity [47]. Due to the growing obesity prevalence, the importance of adequate vitD supplementation appears to be very meaningful in the population of pregnant women living in Serbia. 

Despite the fact that there were no differences in macrosomia rates between the two groups, there were significant differences in the amniotic fluid index (AFI) values. Khanduri et al. suggested that a rise in AFI is the earliest and the most sensitive predictor for GDM [48], a finding that may eventually lead us to conclude that new targeted fasting glucose levels in GDM patients with EGWG should be sought out. The significant differences in PROM prevalence in the EGWG group is most probably related to the AFI rise as well [49]. 

Obesity-induced inflammation, insulin resistance, and β-cell function impairment over time inevitably lead to CVD and DM [50]. Positive family history for CDV and DM implies not only the likelihood that unhealthy lifestyle habits and genetic predisposition are passed from one generation to another, but also a possibility that, during their reproductive age, the parents of our patients might have been obese as well. Maternal obesity and in utero exposure reduces cardiometabolic health in offspring, has long-term consequences, and it is a risk factor for a child’s overweightness and obesity later in life [51,52]. Measures taken during pregnancy can therefore help to break the chain of the vicious circle of heart and metabolic diseases that have trapped generations of families.

APGAR scores in the fifth minute of a newborn’s life were significantly lower in the group of neonates born from mothers with EGWG, and neonates experiencing a reduction in score may be exposed to higher neonatal morbidity [53]. A Canadian study found that the risk of adverse developmental health among children age of five is inversely associated with the first and the fifth minute APGAR scores. Study results have shown that compared with children who had scored nine and ten in the first and the fifth minute of life, children with an APGAR score of nine at both the first and the fifth minute of life had higher rates of developmental vulnerability [54]. Such findings should be seriously considered by physicians working at the obstetrics and neonatology wards, and prompt joint action with physical medicine and rehabilitation specialists ought to be established as soon as possible in order to minimize infant morbidity. The most concerning are the differences in the early motor development between infants whose mothers gained normally and excessively during pregnancy. Pregnant women with EGWG from our study were shown to have offspring with significantly lower scores in psychomotor development measured by AIMS on all occasions from three to twelve months of infant’s life. However, absence of significant decrease in AIMS scores for pronation and supination in the period of twelve months of age might be explained by the fact that these children were without comorbidities and since they were included into psychomotor stimulation treatment, we could assume that due to the mechanisms of neural plasticity [55] particularly in developing brain, there was “catch-up” in developmental milestones. Moreover, our findings demonstrated that women with EGWG are 2.76 times more likely to have offspring with lower AIMS score at three months of age, while at the age of six months such trend decreased to 1.44 times, with further decline to 1.29 times at the age of nine months and then with a slight increase to 1.89 times at the age of twelve months. Our findings are in line with previous reports which stressed that EGWG can affect less favorably neurobehavioral development of newborns [12]. Previous reports stated that fetal brains could be more sensitive to metabolic and nutritional disturbances during pregnancy in comparison to brains of adults [56], therefore, early screening of women with EGWG and continuous follow-up of their newborns is essential in order to facilitate psychomotor development if needed since these infants might be considered to the certain degree at risk for various degrees of neurodevelopmental delay.

Several limitations could be addressed to this study. Individuals in this study belong to Serbia population, and inherited dispositions, as well as specific socio-economic variations, might be existing in different populations. Study sample is an additional limitation, and inclusion of larger group of subjects would increase sensitivity of our findings. Furthermore, the use of metformin as pharmacotherapy treatment in certain patients might be considered as potential limitation to this study as well as the presence of the GDM. Intrauterine exposure to metabolic abnormalities associated with gestational diabetes may induce long term minor neurological defects in the offspring, including motor development vulnerability. Therefore, inclusion of EGWG patients with GDM may impact the interpretation of our results. However, it is not uncommon that EGWG leads to GDM, and it is often challenging to make a homogeneous sample of EGWG patients without GDM.

## 5. Conclusions

EGWG has significantly altered numerous maternal, perinatal, neonatal, and infant parameters in this study. The link between EGWG and adverse neurodevelopmental outcomes in offspring is a complex and multifaceted issue. Our results imply significant deviations in early motor development in the group of infants born from mothers who gained excessively during pregnancy. While research suggests potential associations, the precise mechanisms and causative factors remain areas of ongoing investigation. Further studies are needed to unravel the intricacies of this relationship and inform strategies for preventive interventions and supportive care during pregnancy and infancy. 

## Figures and Tables

**Table 1:** Differences in maternal, perinatal, and neonatal characteristics according to gestational weigh gain (*N* = 200).

Variables		Groups		*p*
		Normal Range GWG (*N* = 113) SV ± SD (95% IP)	Excessive GWG (*N* = 87) SV ± SD (95% IP)	
Maternal age (years)		31.65 ± 4.84 (30.74–32.55)	32.38 ± 4.90 (31.23–33.32)	0.420 *
Pre-pregnancy BMI (kg/m^2^)		21.34 ± 2.41 (20.89–21.79)	26.66 ± 3.14 (25.99–27.33)	<0.001 *
BMI at delivery (kg/m^2^)		26.09 ± 2.47 (25.63–26.55)	33.17 ± 3.48 (32.42–33.91)	<0.001 *
Gestational age (days)		276.74 ± 7.08 (275.42–278.06)	275.03 ± 9.37 (273.04–277.03)	0.325 *
EFW (grams)		3496.81 ± 420.69 (3418.40–3575.23)	3577.64 ± 515.59 (3467.76–3687.53)	0.088 *
AFI (mm)		124.25 ± 33.09 (118.08–130.42)	135.63 ± 45.30 (125.98–145.29)	0.037 *
Pre-pregnancy BMI category	Normal weight	100 (50%)	12 (6%)	<0.001 **
	Overweight	11 (5.5%)	61 (30.5%)	
	Obese	2 (1%)	14 (7%)	
Fetal macrosomia	No	99 (49.5%)	69 (34.5%)	0.112 **
	Yes	14 (7%)	18 (9%)	
Fetal growth restriction	No	105 (52.5%)	80 (40%)	0.797 **
	Yes	8 (4%)	7 (3.5%)	
Family history for CVD	No	97 (48.5%)	62 (31%)	0.011 **
	Yes	16 (8%)	25 (12.5%)	
Family history for DM	No	107 (53.5%)	75 (37.5%)	0.038 **
	Yes	6 (3%)	12 (6%)	
HDP	No	109 (54.5%)	72 (36%)	0.001 **
	Yes	4 (2%)	15 (7.5%)	
GDM	No	99 (49.5%)	50 (25%)	<0.001 **
	Yes	14 (7%)	37 (18.5%)	
GA	No	76 (38%)	37 (18.5%)	<0.001 **
	Yes	37 (18.5%)	50 (25%)	
VitD deficiency	No	54 (27%)	21 (10.5%)	0.001 **
	Yes	59 (29.5%)	66 (33%)	
Metformin use during pregnancy	No	107 (53.5%)	75 (37.5%)	0.038 **
	Yes	6 (3%)	12 (6%)	
Delivery mode	SV	78 (39%)	51 (25.5%)	0.474 **
	CS	19 (9.5%)	20 (10%)	
	Assisted	8 (4%)	5 (2.5%)	
	PIL	3 (1.5%)	4 (2%)	
	CSAFLI	5 (2.5%)	7 (3.5%)	
Genitourinary tract infection	No	92 (46%)	74 (37%)	0.497 **
	Yes	21 (10.5%)	13 (6.5%)	
PROM	No	104 (52%)	71 (35.5%)	0.027 **
	Yes	9 (4.5%)	16 (8%)	
Delivery complications	No	102 (51%)	73 (36.5%)	0.178 **
	Yes	11 (5.5%)	14 (7%)	
APGAR score 1 min of life		8.76 ± 0.66 (8.64–8.88)	8.57 ± 0.74 (8.42–8.73)	0.011 *
APGAR score 5 min of life		9.85 ± 0.43 (9.77–9.93)	9.66 ± 0.55 (9.54–9.77)	0.002 *

GWG—Gestational Weight Gain; BMI—body mass index; CVD—cardiovascular disease; DM—diabetes mellitus; HDP—hypertensive disorder of pregnancy; GDM—gestational diabetes mellitus; GA—gestational anemia; EFW—estimated fetal weight; AFI—amniotic fluid index; PROM—premature rupture of membranes; SV—spontaneous vaginal; CS—cesarean section; PIL—prostaglandins induced labor; CSAFLI—Cesarean section after failed labor induction; * Mann–Whitney U test; ** Chi square test.

**Table 2:** Regression analysis of maternal, pregnancy, and neonatal characteristics according to gestational weigh gain (*N* = 200).

Variables	Univariate Logistic Regression Analysis (Excessive GWG and Normal Range GWG)		
	Exp(B)	95% IP	*p*
Maternal age (years)	1.027	0.969–1.089	0.364
Pre-pregnancy BMI (kg/m^2^)	1.826	1.566–2.129	<0.001
BMI category	27.605	12.635–60.314	<0.001
Gestational age (days)	0.974	0.941–1.009	0.147
Fetal macrosomia	1.845	0.860–3.957	0.116
Fetal growth restriction	1.148	0.400–3.299	0.797
Family history for CVD	2.445	1.209–4.941	0.013
Family history for DM	2.853	1.025–7.940	0.045
HDP	5.677	1.811–17.793	0.003
GDM	5.233	2.591–10.567	<0.001
GA	2.776	1.556–4.952	0.001
VitD deficiency	2.877	1.556–5.317	0.001
Metformin use during pregnancy	2.853	1.025–7.940	0.045
Genitourinary infection	0.770	0.361–1.640	0.497
Delivery mode	1.203	0.939–1.542	0.144
PROM	2.604	1.090–6.219	0.031
Delivery complications	1.778	0.764–4.140	0.182
EFW	1.000	1.000–1.001	0.224
AFI	1.008	1.000–1.015	0.047
APGAR score 1 min of life	0.681	0.452–1.027	0.067
APGAR score 5 min of life	0.434	0.236–0.799	0.007

GWG—Gestational Weight Gain; BMI—body mass index; CVD—cardiovascular disease; DM—diabetes mellitus; HDP—hypertensive disorder of pregnancy; GDM—gestational diabetes mellitus; GA—gestational anemia; EFW—estimated fetal weight; AFI—amniotic fluid index; PROM—premature rupture of membranes.

**Table 3:** Differences in the early motor development scored by AIMS according to gestational weigh gain (*N* = 200).

Variables	Groups		*p*
	Normal Range GWG (*N* = 113) SV ± SD (95% IP)	Excessive GWG (*N* = 87) SV ± SD (95% IP)	
AIMS pronation 3 months	2.74 ± 0.46 (2.66–2.83)	2.39 ± 0.60 (2.26–2.52)	<0.001 *
AIMS supination 3 months	2.84 ± 0.39 (2.77–2.91)	2.41 ± 0.60 (2.29–2.54)	<0.001 *
AIMS total 3 months	5.59 ± 0.73 (5.46–5.73)	4.80 ± 0.99 (4.59–5.01)	<0.001 *
AIMS pronation 6 months	15.73 ± 0.48 (15.64–15.82)	15.32 ± 0.66 (15.18–15.46)	<0.001 *
AIMS supination 6 months	8.76 ± 0.54 (8.66–8.86)	8.25 ± 0.85 (8.07–8.43)	<0.001 *
AIMS sitting 6 months	6.77 ± 0.67 (6.65–6.89)	6.59 ± 0.80 (6.42–6.76)	0.019 *
AIMS standing 6 months	1.96 ± 0.35 (1.90–2.03)	1.91 ± 0.33 (1.84–1.98)	0.256 *
AIMS total 6 months	33.23 ± 1.67 (32.92–33.54)	32.07 ± 1.94 (31.66–32.48)	<0.001 *
AIMS pronation 9 months	19.33 ± 0.66 (19.20–19.45)	18.98 ± 0.81 (18.81–19.15)	0.002 *
AIMS supination 9 months	8.96 ± 0.21 (8.92–8.99)	8.87 ± 0.33 (8.80–8.94)	0.034 *
AIMS sitting 9 months	10.16 ± 0.87 (10.00–10.32)	9.63 ± 1.00 (9.42–9.85)	<0.001 *
AIMS standing 9 months	4.37 ± 0.66 (4.25–4.49)	4.01 ± 0.79 (3.84–4.18)	0.001 *
AIMS total 9 months	42.81 ± 2.06 (42.43–43.20)	41.49 ± 2.48 (40.97–42.02)	<0.001 *
AIMS pronation 12 months	21.00 ± 0.00 (21.00–21.00)	20.99 ± 0.11 (20.97–21.01)	0.254 *
AIMS supination 12 months	9.00 ± 0.00 (9.00–9.00)	9.00 ± 0.00 (9.00–9.00)	1.000 *
AIMS sitting 12 months	11.95 ± 0.26 (11.90–12.00)	11.84 ± 0.43 (11.75–11.93)	0.019 *
AIMS standing 12 months	15.65 ± 0.62 (15.54–15.77)	15.22 ± 0.80 (15.05–15.39)	<0.001 *
AIMS total 12 months	57.60 ± 0.79 (57.46–57.75)	57.05 ± 1.10 (56.81–57.28)	<0.001 *

GWG—Gestational Weight Gain; AIMS—Alberta infant motor scale; * Mann-Whitney U test.

**Table 4:** Regression analysis of the early motor development scored by AIMS according to gestational weigh gain (*N* = 200).

Variables	Univariate Logistic Regression Analysis (Excessive GWG and Normal Range GWG)		
	Exp(B)	95% IP	*p*
AIMS pronation 3 months	0.291	0.166–0.509	<0.001
AIMS supination 3 months	0.187	0.099–0.351	<0.001
AIMS total 3 months	0.362	0.252–0.520	<0.001
AIMS pronation 6 months	0.288	0.169–0.490	<0.001
AIMS supination 6 months	0.319	0.194–0.526	<0.001
AIMS sitting 6 months	0.708	0.479–1.047	0.084
AIMS standing 6 months	0.613	0.267–1.409	0.249
AIMS total 6 months	0.694	0.581–0.829	<0.001
AIMS pronation 9 months	0.521	0.351–0.773	0.001
AIMS supination 9 months	0.320	0.107–0.958	0.042
AIMS sitting 9 months	0.547	0.397–0.754	<0.001
AIMS standing 9 months	0.500	0.334–0.750	0.001
AIMS total 9 months	0.773	0.678–0.883	<0.001
AIMS pronation 12 months	<0.001	<0.001	1.000
AIMS supination 12 months	-	-	-
AIMS sitting 12 months	0.385	0.154–0.961	0.041
AIMS standing 12 months	0.428	0.283–0.649	<0.001
AIMS total 12 months	0.529	0.379–0.736	<0.001

GWG—Gestational Weight Gain; AIMS—Alberta infant motor scale.

## Data Availability

All data are presented in this study. Original data are available on reasonable request from the corresponding author.

## Supplementary Materials

The following supporting information can be downloaded at: https://www.mdpi.com/article/10.3390/jcm13020445/s1. Supplementary Material: Multivariate logistic regression analysis (Excessive GWG and Normal range GWG) of tested study parameters.
