# Supplementary material for: Gestational Weight Gain, Pregnancy Related Complications and the Short-Term Risks for the Offspring
Source: J Clin Med. 2024 Jan 13;13(2):445. doi: 10.3390/jcm13020445 (PMC10816050; doi:10.3390/jcm13020445)
Supplement: Supplementary file 1 [file jcm-13-00445-s001.zip › jcm-2775182-supplementary.pdf]

Supplementary material

Parameters	Multivariate Logistic Regression Analysis (Excessive GWG and Normal range GWG)		
	Exp(B)	95% IP	P
Pre-pregnancy BMI	1.447	0.969-2.161	0.071
BMI category	8.158	0.837-79.519	0.071
Family history for CVD	1.452	0.330-6.387	0.622
Family history for DM	1.116	0.102-12.253	0.929
HDP	0.577	0.093-3.572	0.554
GDM	1.713	0.272-10.800	0.567
GA	3.446	1.091-10.879	0.035
VitD deficiency	2.161	0.642-7.269	0.213
Metformin use during pregnancy	0.175	0.016-1.896	0.152
PROM	3.591	0.773-16.692	0.103
AFI	0.994	0.978-1.011	0.491
APGAR score 5. minute of life	0.661	0.227-1.924	0.448
AIMS pronation 3 months	>100	0.000	1.000
AIMS supination 3 months	>100	0.000	1.000
AIMS total 3 months	0.000	0.000	1.000
AIMS pronation 6 months	1.471	0.031-68.943	0.844
AIMS supination 6 months	0.796	0.074-8.541	0.850
AIMS total 6 months	0.676	0.235-1.950	0.469
AIMS pronation 9 months	2.033	0.628-6.584	0.237
AIMS supination 9 months	11.418	0.607->100	0.104

AIMS sitting 9 months	1.126	0.360-3.524	0.839
AIMS standing 9 months	1.632	0.377-7.071	0.513
AIMS sitting 12 months	>100	0.000	1.000
AIMS standing 12 months	>100	0.000	1.000
AIMS total 12 months	0.000	0.000	1.000

GWG – Gestational Weight Gain; BMI - body mass index; CVD - cardiovascular disease; DM - diabetes mellitus; HDP - hypertensive disorder of pregnancy; GDM - gestational diabetes mellitus; GA - gestational anaemia; AFI - amniotic fluid index; PROM - premature rupture of membranes; AIMS - Alberta infant motor scale
